# Cancer pulmonaire: parcours de soins au service de radiothérapie à l'institut national d'oncologie de Rabat

**DOI:** 10.11604/pamj.2015.21.253.6627

**Published:** 2015-08-06

**Authors:** Amine Lachgar, Nadir Sahli, Ahmedou Toulba, Tayeb Kebdani, Noureddine Benjaafar

**Affiliations:** 1 Institut National d'Oncologie, Service de Radiothérapie, Rabat, Maroc

**Keywords:** Cancer du poumon, radiothérapie, Perdus de vue, lung cancer, radiotherapy, lost to follow-up

## Abstract

L'objectif de cette étude est d'expliquer la discordance entre le nombre important de patients présentant un cancer du poumon localement avancé demandeurs de consultations en service de radiothérapie et le faible nombre de patients effectivement traité. Il s'agit d'une étude décrivant le circuit de soins des patients admis au service de radiothérapie de l'Institut national d'oncologie de Rabat entre le premier mars 2011 et le 29 février 2012 pour la prise en charge d'un cancer du poumon inopérable et/ou non résécable. On a utilisé pour la collecte des données les dossiers cliniques, le registre des nouveaux patients du bureau des admissions de l'institut ainsi que les registres des rendez-vous de consultation et de traitement du service de radiothérapie. 117 patients ont été collectés. Le stade de la maladie n'a pu être déterminé que chez 102 patients, on a ainsi trouvé 53 cancers non métastatiques et 49 cancers métastatiques. Chez les patients avec un cancer non métastatique une radiothérapie palliative a été réalisée chez 9 patients, chez 2 patients la radiothérapie a été contre indiquée, une chimiothérapie néo-adjuvante a été réalisée chez 7 patients et la radio-chimiothérapie concomitante d'emblée fut proposée à 35 patients, mais 34 patients seulement ont pu avoir leur première séance de radiothérapie à visée curative. Cette étude nous a permis de décrire le circuit de soins de nos patients en repérant les points critiques, auxquels on propose des mesures correctives.

## Introduction

Au Maroc, à l'instar de la grande majorité des pays du monde, le cancer du poumon est le premier cancer chez l'homme [1–3]. Les estimations les plus récentes sont fournies par le registre des cancers de la ville de Rabat où l'incidence standardisée du cancer pulmonaire était de 24,8 pour 10000 hommes pour la période 2006-2008 [3]. Il représente aussi la première cause de décès par cancer dans le monde car son pronostic est sombre et le diagnostic est souvent porté à un stade tardif [1]. Le service de radiothérapie de l'Institut national d'oncologie de Rabat au Maroc reçoit annuellement une centaine de patients atteints d'un cancer de poumon mais le quart seulement de ces malades accède au traitement par une irradiation menée à titre curatif. Etant donné que la traçabilité des actes offerts au sein de notre service est assurée à plusieurs niveaux de la demande de soins jusqu'au traitement nous avons essayé de décrire le parcours de soins de ces patients afin d'expliquer la discordance entre le nombre de patients demandeurs des consultations de radiothérapie et le nombre de patients effectivement traités. Les résultats de cette étude constituent une autoévaluation de la qualité des soins au sein de notre service et peuvent orienter la prise de décisions visant à optimiser la prise en charge de nos patients.

## Méthodes

Nous avons réalisé une étude rétrospective en recueillant l'ensemble des patients admis à l'Institut national d'oncologie (INO) entre le premier mars 2011 et le 29 février 2012 adressés au service de radiothérapie pour prise en charge d'un cancer du poumon primitif inopérable et ou non résécable en excluant les malades avec une preuve de métastase à distance adressés pour radiothérapie palliative. Pour la collecte des données on a utilisé en plus des dossiers cliniques des patients, le registre des nouveaux malades du bureau des admissions de l'institut, le registre des rendez-vous de consultation et de programmation du traitement au service de radiothérapie, et le système informatique d'enregistrement et de vérification de traitement. Un support informatique et un autre en papier ont été utilisés. Le support papier a été utilisé sous la forme d'un feuillet propre à chaque patient traçant son circuit de soins. Le logiciel de gestion des tableaux Excel a été utilisé comme support informatique permettant le recueil de l'ensemble des variables présentés dans le chapitre des résultats.

## Résultats

Au total, 117 patients atteints d'un cancer pulmonaire ont été collectés.

### Description de la population

Sur les 117 patients il y avait une nette prédominance masculine: 111 hommes (95%) et 6 femmes (5%). L’âge médian des personnes incluses dans cette étude était de 57 ans, avec des extrêmes allant de 36 à 91 ans, les 6 femmes de cette étude étaient toutes âgées de plus de 70 ans. Facteur pronostic essentiel le performance status initial de ces malades n'a été retrouvé que dans 45% des dossiers médicaux. Parmi les cas renseignés, 31% des patients avaient une activité normale au moment du diagnostic, plus de la moitié avaient une activité réduite mais restaient autonomes et 14% des patients nécessitaient une assistance. Les types histologiques par ordre de fréquence décroissant étaient le carcinome épidermoïde dans 49% des cas, l'adénocarcinome dans 44% des cas et le carcinome à grande cellule chez 7% de malades.

### Description du circuit de prise en charge

le circuit de prise en charge de nos patients est segmenté en plusieurs étapes:

#### Admission des malades

Un bureau d'accueil de l'institut entre le 1er mars 2011 et le 29 février 2012, 117 nouveaux patients étaient admis pour traitement d'un cancer du poumon non à petites cellules sans preuve de métastases à distance et orientés vers le service de radiothérapie. Chez la majorité des malades le bilan d'extension se limitait au bilan d'extension locorégionale à base d'un scanner thoracique. Le bilan initial des malades a été vérifié lors de l'ouverture du dossier et les examens paracliniques manquants (scanner cérébral et/ou scintigraphie osseuse et/ou échographie ou scanner abdominal) ont été demandés si nécessaire. Un rendez-vous pour une première consultation en service de radiothérapie a été accordé à tous ces malades.

#### Consultation au service de radiothérapie

Moment clé du parcours thérapeutique, elle a pour but de confirmer l'indication de la radiothérapie et sa faisabilité. 111 est le nombre de patients qui ont été orientés vers la consultation de radiothérapie et l'ont eu effectivement car 6 patients (5%) étaient perdus de vue dés leurs admission à l'institut. Au premier rendez-vous de consultation en radiothérapie 19% seulement de ces malades avaient un bilan complet et la majorité des malades a eu un ou plusieurs passages par la consultation avant de compléter un bilan d'extension permettant de proposer une décision thérapeutique. Une fois complété le bilan d'extension a permis de préciser le stade de la maladie chez 102 patients car 9 malades étaient perdus de vu au cours de cette étape de prise en charge pré-thérapeutique. En classant en stade les cas de notre série selon la 7^éme^ édition de la classification TNM des tumeurs malignes on a trouvé [4]: 53 cancers non métastatiques avec 4 patients avec un cancer pulmonaire stade IIB, 23 patients avec un cancer du poumon stades IIIA, et 26 patients avec un cancer pulmonaire stade IIIB. 49 cancers métastatiques: les métastases cérébrales, pulmonaires et osseuses étaient les plus fréquentes avec 18 métastases cérébrales, 11 métastases au niveau du poumon et/ou de la plèvre, 8 métastases osseuses, 4 cas avec localisation hépatique et 8 métastases multiples.

#### Proposition thérapeutique

Pour les 53 malades avec une maladie non métastatique on avait proposé une radiochimiothérapie concomitante d'emblée chez 35 malades et après une chimiothérapie néo-adjuvante chez 7 patients. Une radiothérapie palliative a été réalisée chez 9 patients, et chez 2 patients la radiothérapie a été contre indiquée à cause d'une insuffisance respiratoire chez un malade et une cardiopathie ischémique grave chez l'autre. Les malades avec une maladie métastatique ont étaient orientés vers la consultation d'oncologie médicale.

#### Accès à la première séance de radiothérapie

Le suivi des malades chez qui un traitement à visée curative a été retenu a montré que parmi les malades programmés à recevoir une radiochimiothérapie d'emblée 3 patients étaient perdu de vue avant le traitement, et parmi les 7 malades ayant reçu une chimiothérapie néo-adjuvante 4 malades ont développé des métastases à distance avant la fin des cures programmées et un malade fut perdu de vue après la deuxième cure ainsi 2 malades seulement ont pu effectivement bénéficier d'une radiochimiothérapie concomitante après la chimiothérapie néo-adjuvante. Au totale sur les 117 patients inclus initialement dans cette série 34 seulement (29%) ont pu avoir leur première séance de radiothérapie dans le cadre d'une radiochimiothérapie concomitante.

## Discussion

Les études concernant le parcours de soins des patients atteints de cancer sont des études visant à décrire le processus de prise en charge de ces malades au sein d'une formation sur une période donnée [5]. Dans la présente étude, nous avons essayé de décrire les modes de prise en charge des malades atteints d'un cancer du poumon au sein de notre service ce qui nous a permis de repérer les principaux points critiques, et de proposer ainsi des mesures correctives visant à garantir une gestion optimale de nos malades. L'absence d'une sélection optimale des malades est un problème majeur rencontré lors de cette étude car un pourcentage très élevé de malades métastatiques atteignant 42% de l'ensemble de la population a été retrouvé ce qui est expliqué par le diagnostic tardif de ce cancer au Maroc [6]. L'absence d'un thésaurus régional est un autre obstacle essentiel, l'objectif d'un tel référentiel est d'homogénéiser les pratiques de prise en charge des patients atteints d'un cancer du poumon en établissant des recommandations pratiques concernant l'ensemble des étapes de cette prise en charge et en veillant à diffuser ces directives parmi les médecins ayant des activités en cancérologie thoracique [7]. La quasi inexistence des réunions de concertation pluridisciplinaire vient aggraver la situation, ces réunions sont un élément essentiel dans l'organisation des soins en cancérologie, ils doivent réunir des médecins de différentes spécialités impliquées dans le traitement du cancer du poumon: pneumologues, chirurgiens thoraciques, oncologues radiothérapeutes, oncologues médicaux, radiologues et anatomopathologistes [8]. Au cours de ces réunions, le dossier du patient doit être discuté de façon collégiale, avec une évaluation des bénéfices et des risques de chaque traitement. Un autre problème tout aussi important est celui des perdus de vue, ainsi dans cette étude 18 patients (15%) étaient perdus de vue avant même la phase de traitement. Pour déterminer les causes de ce taux élevé on a essayé de rechercher activement les malades perdus de vue par des appels téléphoniques. Si 7 patients sont restés injoignables, 11 ont pu être retrouvés, parmi eux 8 étaient décédés, et 3 étaient suivis par d'autres centres de traitement, mais le caractère rétrospectif de l’étude ne nous permet pas de préciser si le décès de ces malades est une cause ou une conséquence du fait d’être perdu de vue. Vu la pauvreté des informations concernant ces patients nous avons été amenés à formuler des hypothèses sur les causes du nombre important des malades perdus de vue: le diagnostic d'une proportion élevée des patients à un stade avancé de leur maladie; l’état général médiocre de la plupart des patients. La forte mortalité de ce cancer; le manque d'appui social adéquat sur les sites de prise en charge; le faible niveau socio-économique de la majorité des malades. L'importance des frais de transport et d'habitat pour les patients éloignés. Ainsi l'identification de ces patients à risque va permettre de proposer une prise en charge adaptée à leur contexte en essayant de personnaliser davantage les traitements et de modifier l'ordre des patients dans les listes d'attente des rendez-vous de consultation et de traitement au sein de notre service.

Un autre objectif principal des études concernant les parcours de soins des malades atteints de cancers est de mesurer le degré d'intégration des résultats des différents essais cliniques dans la pratique quotidienne du centre [9]. Le standard thérapeutique dans le traitement des cancers du poumon non à petites cellules localement avancés inopérables est la radio-chimiothérapie concomitante [10, 11]. Dans notre série 34 patients (65%) parmi 52 ont bénéficié de ce traitement ce qui signifie qu'il y a place à l'amélioration de la prise en charge de ces patients dans notre institut. Dans notre série 7 patients (13,5%) parmi les 52 patients avec un cancer du poumon localement avancé ont bénéficié d'une chimiothérapie néo-adjuvante mais seulement 2 patients ont pu avoir une radio-chimiothérapie concomitante par la suite. A ce jour il n'y a pas de preuve que la chimiothérapie d'induction avant la radio-chimiothérapie concomitante apporte un bénéfice [12]. Donc il parait nécessaire de limiter l'indication de la chimiothérapie néo-adjuvante aux cas où l'irradiation thoracique sera très toxique du fait d'un gros volume tumoral. Le dernier point concerne la radiothérapie chez les sujets âgés. L’évolution de la démographie rend de plus en plus importante la question du traitement optimal du sujet âgé. Dans le groupe de malades ayant reçu une radiothérapie palliative la moyenne d’âge était de 65,5 ans et tous ces malades étaient âgés de plus de 60 ans. Alors est ce que l’âge peut constituer un motif suffisant pour proposer un traitement sub-optimal’. Peu de données sont disponibles dans la littérature pour répondre à cette question, les données qui existent sont établies à partir de patients très sélectionnés qui ne sont pas représentatifs de la population générale des sujets de plus de 70 ans. Mais la majorité des études s'accorde sur le fait que l’âge ne représente pas un facteur limitant à l'administration de la radiothérapie à visée curative chez le sujet âgé, atteint d'un cancer bronchique inopérable et ou non résécable néanmoins une évaluation gériatrique au préalable reste indispensable [13].

## Conclusion

La prise en charge des cancers bronchiques nécessite des interventions pluri-disciplinaires et multi-sites qu'il convient de coordonner, standardiser et surtout évaluer régulièrement. Cette étude a permis de décrire le circuit de soins des patients atteints d'un cancer bronchique primitif au sein de notre service tout en valorisant les bonnes pratiques organisationnelles et en repérant les principaux points critiques, auxquels on propose des mesures correctives.
